# Towards the diagnosis of dengue virus and its serotypes using designed CRISPR/Cas13 gRNAs

**DOI:** 10.6026/97320630018661

**Published:** 2022-08-31

**Authors:** Archana Prajapati, Ashmita Tandon, Vikrant Nain

**Affiliations:** 1 School of Biotechnology, Gautam Buddha University, Greater Noida-201312, Uttar Pradesh, India

**Keywords:** Clustered Regularly Interspaced Short Palindromic Repeats (CRISPR), Cas13, Dengue virus, serotypes, diagnosis, gRNA pool, secondary structure, free energy

## Abstract

Dengue Virus (DENV) is a mosquito-borne virus that is prevalent in the world's tropical and subtropical regions. Therefore, early detection and surveillance can help in the management of this disease. Current diagnostic methods rely primarily on ELISA, PCR, and RT-PCR, among others, which can only be performed in specialized laboratories and require sophisticated instruments and technical expertise. CRISPR-based technologies on the other hand have field-deployable viral diagnostics capabilities that could be used in the development of point-of-care molecular diagnostics. The first step in the field of CRISPR-based virus diagnosis is to design and screen gRNAs for high efficiency and specificity. In the present study, we employed a bioinformatics approach to design and screen DENV CRISPR/Cas13 gRNAs for conserved and serotype-specific variable genomic regions in the DENV genome. We identified one gRNA sequence specific for each of the lncRNA and NS5 regions and identified one gRNA against each of DENV1, DENV2, DENV3, and DENV4 to distinguish the four DENV serotypes. These CRISPR/Cas13 gRNA sequences will be useful in diagnosing the dengue virus and its serotypes for in vitro validation and diagnostics.

## Background:

Dengue is a major arthropod-borne viral disease caused by the dengue virus (DENV), which is widespread in tropical and subtropical regions of the world and is spread by the Aedesaegyptis and Aedesalbopictus mosquitos [1]. Its clinical manifestations range from mild Dengue Fever (DF) to severe Dengue Haemorrhagic Fever (DHF) and Dengue shock syndrome (DSS) [1]. Dengue virus (DENV) is an enveloped, positive-sense, ss-RNA virus of the Flaviviridae family and the genus Flavivirus [2], [3]. DENV has four well-known serotypes (DENV1, DENV2, DENV3, DENV4) based on antigen cross-reactivity, with each serotype having distinct genotypes [2][3]. The DENV genome (∼11 kb) comprises one open reading frame (ORF) flanked by 5' and 3' UTR regions [2] [4]. Its ORF encodes a single polyprotein, which is cleaved post-transactionally to generate three structural proteins: capsid (C), pre-membrane/membrane (prM/M), and envelope (E), as well as seven non-structural (NS) proteins (NS1, NS2A, NS2B, NS3, NS4A, NS4B, and NS5)(Figure 1 - see PDF) [1]. There are currently no particular antiviral medicines or vaccinations available to treat or prevent dengue infection [2], [5],[6]. The sole alternative that relies on an early and accurate dengue diagnosis is symptomatic and supportive therapy of the disease. Dengue infection is diagnosed through the isolation and characterization of the DENV virus, serological tests that detect DENV-related antigens and/or antibodies in patient plasma/serum, and molecular diagnosis [4], [5], [7]. The DENV nucleic acid (RNA) is detected using an isothermal nucleic acid sequence-based amplification assay (NASBA), a reverse transcriptase-polymerase chain reaction (RT-PCR) (one-step or nested RT-PCR assay), and a real-time RT-PCR assay; these molecular methods require skilled technicians and specialized laboratories [4] [5] [8]. As a result, more precise and point-of-care (POC) molecular approaches that enable improved clinical treatment, proper disease management, surveillance, and prevention of dengue outbreaks are required. Clustered Regularly Interspaced Short Palindromic Repeat (CRISPR), is a highly variable locus consisting of short and identical repeated palindromic sequences that are naturally found in the genome of bacteria, separated by large spacer sequences[9]. The CRISPR locus is transcribed to generate pre-crRNA, which is then processed by CRISPR-associated (Cas) proteins to generate mature CRISPR guide RNA (gRNAs) [10]. CRISPR-associated (Cas) proteins specifically act as RNA-guided endonucleases which together with gRNA induce indiscriminate cleavage of target nucleic acid thereby protecting the bacterial cell against any invading bacteriophage [9], [10]. CRISPR-Cas-based diagnosis relies on the identification of viral nucleic acid sequences by gRNAs followed by non-specific cleavage of viral nucleic acid by Cas [11], [12]. This reactionis usually coupled with probe molecules that generate a fluorescent/visual signal which is detected [11], [12]. SHERLOCK (Specific High Sensitivity Enzymatic Reporter Unlocking), DETECTR (DNA endonuclease-targeted CRISPR transReporter),CARVER (Cas13-assisted restriction of viral expression and readout), PAC-MAN (Prophylactic Antiviral CRISPR in human cells), SHINE (Streamlined Highlighting of Infections to Navigate Epidemics), All-In-One Dual CRISPR-Cas12a (AIOD-CRISPR)are examples of CRISPR-Cas molecular methods used in disease diagnosis [13][15]. In this study, we have designed DENV-specific CRISPR-Cas13 gRNAs for the conserved and variable/hyper variable genomic regions among four DENV serotypes using bioinformatics tools. These CRISPR-Cas13 gRNA sequences serve as molecular detectors that provide point-of-care molecular diagnosis useful in the detection and clinical evaluation of the dengue virus and its serotypes.

## Methodology:

The dataset of complete genome sequences of four Dengue virusserotypesDENV1 (NC_001477), DENV2 (NC_001474), DENV3 (NC_001475), DENV4 (NC_002640) were compiled from the Dengue Virus Variation Resource Centre at National Centre for Biotechnology Information (NCBI) [16]. The Multiple Sequence Alignment (MSA) of these sequences was performed using Bio Edit v 7.2 [17]. The CRISPR-Cas13 gRNAs were designed the using CHOPCHOP v2 CRISPR designing web tool [18]. These gRNA sequences were visually inspected for the number of mismatches and number of bases conserved to obtain potentialCRISPR-Cas13 gRNA sequences showing high specificity (maximum target annealing) and low off-target selectivity (minimum off-target binding) for the conserved and variable portions in the DENV genome. The secondary structures and free energies of gRNA sequences were predicted using the default parameters in RNA fold web-server [19].

## Results and Discussion:

We identified the conserved and variable sequences across the genome of four common DENV serotypes. Subsequently, these selected genomic regions were used to design CRISPR-Cas13gRNAsequences.

## Sequence retrieval and multiple sequence alignment:

The extensive whole-genome Multiple sequence alignment (MSA) of four DENV reference serotypes DENV1 (Gen Bank: NC_001477.1), DENV2 (Gen Bank: NC_001474.2), DENV3 (Gen Bank: NC_001475.2), and DENV4 (Gen Bank: NC_002640.1) revealed lncRNA and NS5 genomic regions that are conserved across all DENV serotypes, while variable sequences found in the NS2A and NS2B gene. The NS2A gene is involved in coordination during RNA packaging and replication whereas the NS2B gene serves as a co-factor in the structural activation of DENV serine protease NS3 [1]. NS2B gene also assists in viral replication and blocks IFN- induced signal transduction [1]. Gene NS5 codes for methyltransferase domain (located at residues 1-269 amino acids) and RNA-dependent RNA polymerase (located at residue 270-900 amino acids) [1].

## Designing of CRISPR/Cas13 gRNAs against conserved regions in DENV genome:

MSA of reference DENV serotypes revealed that the most conserved sequences are in the NS5 and long non-coding RNA (lncRNA) regions (Figures 2 and 3 - see PDF). As a result, genomic sequences of the DENV2 (Gen Bank: NC_001474.2) lncRNA (0.425 kb) and NS5 (2.7 kb) regions were used as a query in the CHOPCHOP tool to design CRISPR/Cas13 gRNAs for Dengue diagnosis. The CHOPCHOP online tool returned 293 gRNA sequences for DENV lncRNA and 1973 gRNAs for NS5. These gRNA sequences were visually inspected for the number of conserved nucleotides in the MSA, and the sequences with the highest number of conserved nucleotides among four DENV serotypes were selected, yielding a total of 251 gRNA sequences for lncRNA and 268 gRNA sequences for NS5. To narrow the search even further and to improve gRNA specificity against the target, gRNA sequences were re-examined for the number of sequence mismatches in the lncRNA and NS5 regions, and the gRNA sequences with the least mismatches were selected. We selected a total of 17 lncRNA-specific sequences by selecting gRNA sequences with a mismatch of less than or equal to two nucleotides and a total of 10 NS5 specific gRNA sequences by selecting gRNA sequences with a mismatch of less than or equal to five nucleotides because lncRNA was found to be more conserved among DENV serotypes than NS5, as indicated by MSA. Furthermore, gRNA sequences with no mismatches in the core region, i.e. within a stretch of 11-18nts, were highly target-specific and anneal quickly with complementary sequences in the target genome [20]. As a result, we visually inspected the lncRNA and NS5 specific gRNA sequences for mismatches and eliminated those with mismatches within the core region. Our findings showed that 11 of the 17 lncRNA gRNA sequences have no mismatches within an 8-base sequence stretch spanning 11-18 nucleotides, while 4 of 10 NS5 gRNA sequences have only one mismatch within the central seed region (Table 1 - see PDF).

## Prediction of gRNA secondary structure:

There is a link between the secondary structure of gRNA and gene editing efficiency. The formation of secondary structures in gRNA has a significant impact on cleavage efficiency. More specifically, when the Gibbs free energy (ΔG) for the formation of the most stable RNA structure is between -2 and 0 kJmol-1, cleavage efficiencies are highest [21]. As a result, the gRNA can invade the target strand without the aid of the gRNA scaffold. RNA fold Web Server was used to evaluate the secondary structures and minimum free energy of lncRNA and NS5 specific gRNA sequences (Table 1 and Figure 4 - see PDF). We selected two DENV-specific gRNAs based on the minimum free energy, least number of mismatches among DENV serotypes, zero self-complementarity of gRNA, GC content, and no mismatch within the central region and gRNA secondary structure. One sequence (TAGAGGAGACCCCCCCGAAACAAAAAAC) is specific for targeting the lncRNA and the other (ATGTATGCCGATGACACCGCAGGATGGG) is specific for targeting the NS5 gene (Table 1 and Figure 4 - see PDF). These two CRISPR-Cas13 gRNA sequences have a low possibility of self-annealing; allowing them to recognize and bind to the target site within the DENV genome's conserved regions.

## Designing of serotype-specificCRISPR/Cas13 gRNAs:

Using reference genomic sequences of the NS2A and NS2B genes from four DENV serotypes, the CHOPCHOP online tool identified a total of 3,072 CRISPR-Cas13 gRNA sequences, with 776, 772, 763, and 761 gRNA sequences for the NS2A and NS2B regions of DENV1, DENV2, DENV3, and DENV4 respectively. The number of conserved bases in the NS2A and NS2B regions of these gRNA was visually inspected in MSA. The gRNA sequences showing the lowest number of conserved bases among the serotypes was selected to provide the most variable serotype-specific targets. It narrowed down serotype-specific gRNAs to 143, 173, 136, and 275 for DENV1, DENV2, DENV3, and DENV4 respectively. To further reduce the number of gRNA sequences we identified gRNA sequences with the highest specificity and selected only those gRNAs that show the least conserved bases not more than 3 bases among serotypes. This screening further reduced the gRNA sequences to 5, 19, 14, and 11for DENV1, DENV2, DENV3, and DENV4 respectively (Supplementary Table 1, selected gRNA sequences - see PDF).

## Prediction of secondary structure in gRNA:

The secondary structures and minimum free energy of DENV serotype-specific gRNAs were predicted to aid in the selection of gRNAs that are more efficient and specific to target RNA. For the targets DENV1, DENV2, DENV3, and DENV4, we obtained 1, 5, 5, and 2 potential serotype-specific gRNA sequences with zero or nearly zero free energy, respectively (Figure 5 - see PDF). These gRNAs were then screened for minimum free energy, secondary structure, and the fewest number of conserved bases across DENV serotypes. We got one gRNA for each of the DENV serotypes, which were TCAAAACAACTTTTTCATTGCACTATGC, AACCCTCTCAAGAACCAGCAAGAAAAGG, TTAGCTTGAAAGACACACTCAAAAGGAG, and AACAGCACTCATCCTAGGAGCCCAAGCT for DENV1, DENV2, DENV3, and DENV4 respectively (Figure 5 - see PDF, gRNA in bold). These gRNA sequences have the potential for efficient binding with the genome of selected DENV serotypes. CRISPR-based diagnostics are next generation biosensing techniques for detection of viral and bacterial infections. SHERLOCK has been used for detection of Dengue, Zika, bacterial infections, and tumour changes in cell-free DNA using CRISPR-Cas13a [13], [22]. These In-silico designed28nt long gRNAs sequences can detect complementary sequences in the DENV genome. These DENV-specific gRNA sequences coupled with Cas13 enzyme can be used in sequence-specific cleavage of the target genome and reporter molecules which can be developed into molecular test useful in identification of dengue virus and its serotypes. Molecular tests like these provide rapid detection and quick identification of virus in clinical samples from dengue infected patients providing an early and accurate disease diagnosis.

## Conclusions:

Early and serotype specific diagnosis is important in management of spread of the any diseases and treatment of patients. CRISPR-Cas13 based diagnostics are highly sensitive and specific as well as cost-effective, fast, and can be used as point of care diagnostics. The present study gRNA sequences specific for DENV were designed to provide molecular diagnosis. These gRNA will be valuable for in vitro evaluation the sensitivity and efficiency of CRISPR-Cas13-based POC diagnostics.

## List of Abbreviations:

DENV: Dengue virus; ZIKV: Zika virus; ELISA: Enzyme-linked Immunosorbent Assay; PCR: Polymerase Chain Reaction; RT-PCR: reverse transcriptase-polymerase chain reaction; CRISPR: Clustered Regularly Interspaced Short Palindromic Repeat; Cas: CRISPR associated proteins; gRNA: guide RNA; lncRNA: Long non-coding RNA; DF: Dengue Fever; DHF: Dengue Haemorrhagic Fever; DSS: Dengue shock syndrome; ORF: Open Reading Frame; UTR: Untranslated region; C: Capsid; prM/M: pre-membrane/membrane; NS: Non-structural; E: envelope; NASBA: Nucleic acid sequence-based amplification assay; SHERLOCK: Specific High Sensitivity Enzymatic Reporter Unlocking; DETECTR: DNA endonuclease-targeted CRISPR transReporter; CARVER: Cas13-assisted restriction of viral expression and readout; PAC-MAN: Prophylactic Antiviral CRISPR in human cells; SHINE : Streamlined Highlighting of Infections to Navigate Epidemics; AIOD-CRISPR:All-In-One Dual CRISPR-Cas12a; FELUDA: FNCas9 Editor Limited Uniform Detection Assay; MSA: Multiple sequence alignment; IFN: Interferons; ΔG: Gibbs free energy; RAA: recombinase aided amplification; STOP: SHERLOCK testing in one pot; CREST: Cas13-based, rugged, equitable, scalable testing; HPV: Human papillomavirus.
